# Expression of Pumpkin *CmbHLH87* Gene Improves Powdery Mildew Resistance in Tobacco

**DOI:** 10.3389/fpls.2020.00163

**Published:** 2020-04-03

**Authors:** Wei-Li Guo, Bi-Hua Chen, Yan-Yan Guo, Xue-Jin Chen, Qing-Fei Li, He-Lian Yang, Xin-Zheng Li, Jun-Guo Zhou, Guang-Yin Wang

**Affiliations:** ^1^School of Horticulture Landscape Architecture, Henan Institute of Science and Technology, Xinxiang, China; ^2^Henan Province Engineering Research Center of Horticultural Plant Resource Utilization and Germplasm Enhancement, Xinxiang, China

**Keywords:** pumpkin, powdery mildew, *CmbHLH87*, functional analysis, tobacco

## Abstract

Powdery mildew (PM), caused by *Podosphaera xanthii*, is a major threat to the global cucurbit yield. The molecular mechanisms underlying the PM resistance of pumpkin (*Cucurbita moschata* Duch.) are largely unknown. A homolog of the basic helix-loop-helix (bHLH) transcription factor was previously identified through a transcriptomic analysis of a PM-resistant pumpkin. In this study, this bHLH homolog in pumpkin has been functionally characterized. CmbHLH87 is present in the nucleus. *CmbHLH87* expression in the PM-resistant material was considerably downregulated by PM; and abscisic acid, methyl jasmonate, ethephon, and NaCl treatments induced *CmbHLH87* expression. Ectopic expression of *CmbHLH87* in tobacco plants alleviated the PM symptoms on the leaves, accelerated cell necrosis, and enhanced H_2_O_2_ accumulation. The expression levels of *PR1a*, *PR5*, and *NPR1* were higher in the PM-infected transgenic plants than in PM-infected wild-type plants. Additionally, the chlorosis and yellowing of plant materials were less extensive and the concentration of bacteria at infection sites was lower in the transgenic tobacco plants than in the wild-type plants in response to bacterial wilt and scab pathogens. *CmbHLH87* may be useful for genetic engineering of novel pumpkin cultivars in the future.

## Introduction

Pumpkin (*Cucurbita moschata* Duch.) is an important vegetable crop and is widely cultivated in China, with total harvested area of 438,466 hectares in 2017 (i.e., 17.42% of the global area) (Food and Agriculture Organization, 2017). Powdery mildew (PM) is a fungal disease seriously affecting Cucurbitaceae crops yield including cucumber, melon, watermelon, pumpkin, and squash in the world. The disease is mainly caused by *Podosphaera xanthii* (formerly known as *Sphaerotheca fuliginea*), which is a biotrophic plant pathogen (Perez-Garcia et al., 2009; Fukino et al., 2013). Excessive fungicide application poorly control PM, because it not only increases selection pressure on PM pathogens to adapt increasing levels of fungicide resistance, but it also may be harmful for human health and the environment. Therefore, studying the moleculer mechanism of PM by exploiting the resistant genes to breed resistant varieties represents a favored strategy to control PM.

The basic helix-loop-helix (bHLH) transcription factors constitute one of the largest transcription factor families in plants, wherein they help regulate developmental processes and responses to environmental stresses. These proteins have a 60-amino-acid conserved domain, which contains the following two functionally distinct regions: an N-terminal basic region (13–17 amino acids) that functions as a DNA-binding domain and a C-terminal HLH region that contributes to the formation of homodimers or heterodimers (Heim et al., 2003; Toledo-Ortiz et al., 2003). Recent studies have indicated that a number of bHLH transcription factor genes are involved in responses to abiotic stresses including drought, salt, and cold. The overexpression of *AtbHLH068* and *OsbHLH148* in transgenic *Arabidopsis thaliana* and rice, respectively, reportedly induces drought stress tolerance *via* abscisic acid (ABA)– and jasmonic acid (JA)–mediated signaling pathways (Seo et al., 2011; Le Hir et al., 2017). In rice, *OsbHLH062*, *OsJAZ9*, and *OsNINJA* form a transcriptional regulatory complex that fine-tunes the expression of JA-responsive genes involved in salt stress tolerance (e.g., *OsHAK21*) (Wu et al., 2015). Additionally, *VaICE1/VaICE2*, *ZmmICE1*, and *NtbHLH123* are key regulators in the C-repeat binding factor regulatory pathway controlling cold tolerance (Xu et al., 2014; Lu et al., 2017; Zhao et al., 2018). Moreover, bHLHs influence the adaptation and resistance of plants to pathogen stress. An earlier investigation revealed that *OsDPF* expression is induced in the leaves of blast-infected rice plants, and the overexpression and knockdown of *DPF* considerably increases and decreases the accumulation of momilactones and phytocassanes, respectively (Yamamura et al., 2015). The overexpression of wheat *bHLH060* in transgenic *A. thaliana* negatively regulates the resistance to *Pseudomonas syringae* through JA and ethylene (ET) signaling pathways (Wang et al., 2015). A recent study on wheat indicated that the expression levels of 28 and 6 *TabHLH* genes are obviously downregulated and upregulated, respectively, in response to a PM infection (Wei and Chen, 2018). However, bHLH functions related to biotic stress resistance remain poorly characterized in plants.

In our previous study, the PM-resitant pumpkin resources were obtained in an 8-year outdoor field observation study (Zhou et al., 2010). However, the resistant mechanism underlying pumpkin response to biotic stress is not yet elucidated. Thus, a RNA sequencing analysis of PM-infected pumpkin identified 4,716 differentially expressed genes, including gene encoding *bHLH* transcription factor (*bHLH87*) (Guo et al., 2018). The expression of this *bHLH87* homolog in response to PM, hydrogen peroxide (H_2_O_2_), salicylic acid (SA), ABA, methyl jasmonate (MeJA), ethephon (Eth), and NaCl treatments were analyzed through real-time quantitative PCR (RT-qPCR). To evaluate its function in disease resistance, we expressed CmbHLH87 in tobacco ectopically. Transgenic tobacco plants constitutively overexpressing *CmbHLH87* were more resistant to PM, bacterial wilt, and scab than the control plants.

## Results

### Isolation of *CmbHLH87* and Subcellular Localization

Pumpkin PM-related genes were identified in a transcriptome (Guo et al., 2018). One of these clones was 89% identical at the nucleotide level to *C. melo bHLH87*. Full length of this homolog was obtained by a homology-based candidate gene method (named *CmbHLH87*) and submitted to the GenBank database (accession number MH105822). The size of this gene was 1,222 bp, including a 1,068-bp open reading frame (355 amino acids). The predicted polypeptide was relatively acidic, with a pI of 5.96, and an Mw of 39.1 kDa. A phylogenetic tree was conducted between the overall amino acid sequences of CmbHLH87 and other known bHLH proteins. CmbHLH87 was clustered into the bHLH VIII subfamily (Heim et al., 2003) (**Supplementary Figure S1**). An alignment of the deduced CmbHLH87 amino acid sequence with homologous sequences is presented in **Supplementary Figure S2**. At the amino acid level, CmbHLH87 was highly similar to the bHLH87 transcription factors from *C. moschata* (99.1% identical), *Cucurbita pepo* (98.8% identical), and *Cucurbita maxima* (71.0% identical), but was relatively dissimilar to *Nicotiana tabacum* bHLH87 (38.3% identical) and *Arabidopsis* AtbHLH087 (41.7% identical). The predicted amino acid sequence contained a conserved bHLH domain (amino acids 256–305) and a helix-loop-helix structure at the C terminal.

**Figure 1:**
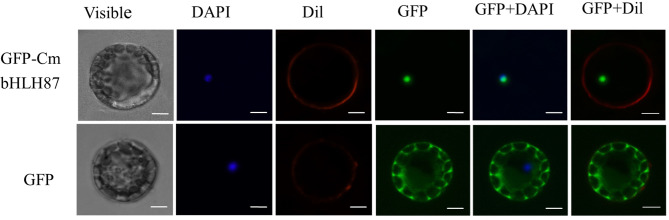
The subcellular localization of pumpkin CmbHLH87. The fused green fluorescent protein (GFP)–CmbHLH87 and alone GFP constructs were introduced into *Arabidopsis* protoplast by polyethylene glycol-mediated protoplast transformation. The fluorescent signals were detected using a confocal fluorescence microscope. Scale bars = 5 μm.

The subcellular localization of CmbHLH87 was assessed with a CmbHLH87-GFP fusion protein into *Arabidopsis* protoplasts under the control of the 35S CaMV promoter. The GFP signal in protoplasts producing GFP alone was detected in the cytoplasm and nucleus (**Figure 1**) supported by Guo et al. (2019), whereas the signal from the CmbHLH87-GFP fusion protein was detected exclusively in the nucleus.

### Expression Patterns of *CmbHLH87* in Response to PM and Exogenous Treatments

**Figure 2:**
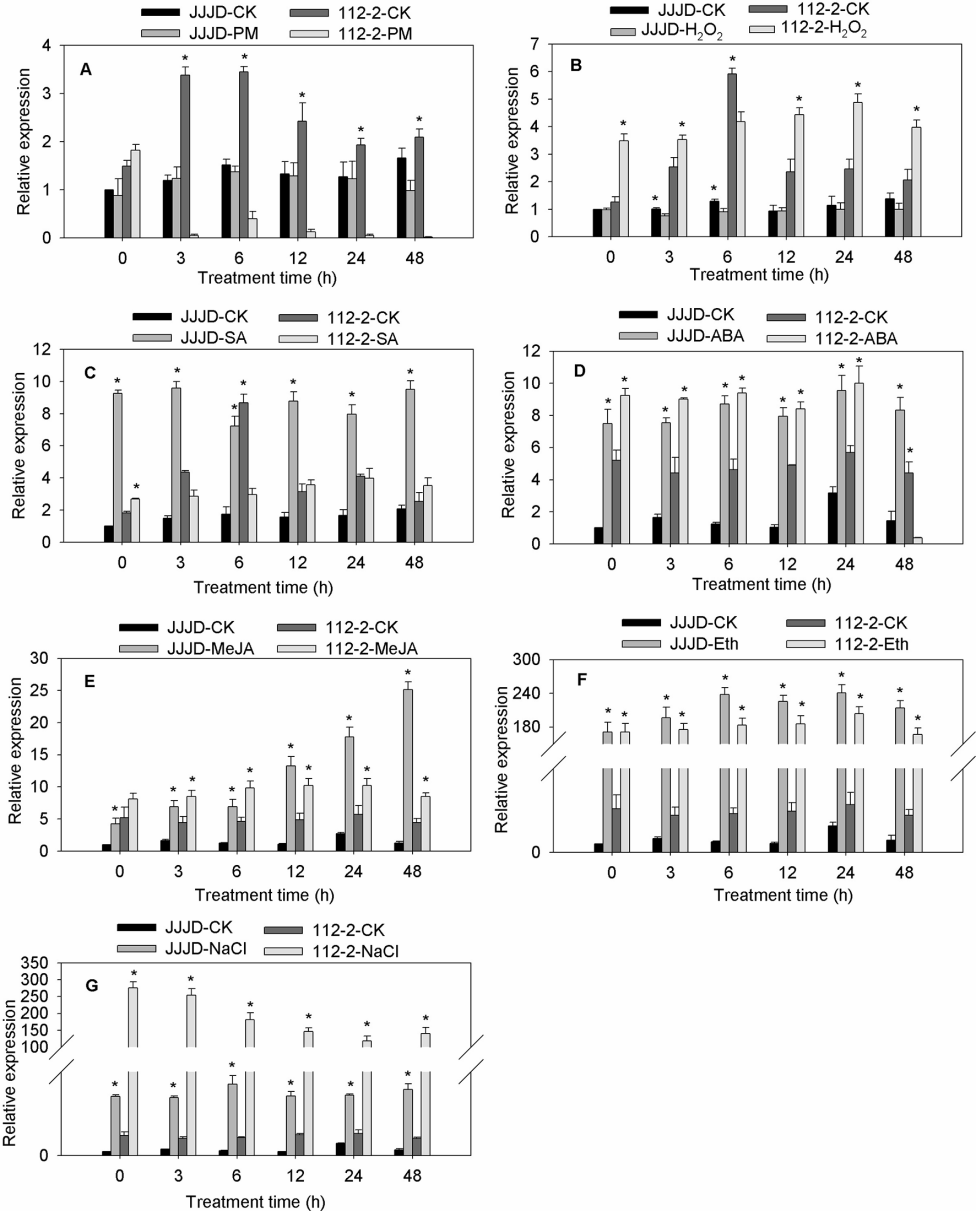
*CmbHLH87* expression in response to powdery mildew and exogenous treatments. The pumpkin seedlings were sprayed with a spore suspension **(A)**, exogenous hydrogen peroxide (H_2_O_2)_ **(B)**, salicylic acid (SA) **(C)**, abscisic acid (ABA) **(D)**, methyl jasmonate (MeJA) **(E)**, ethephon (Eth) **(F)**, and NaCl **(G)**. The *β-actin* gene was used as an internal reference for qRT-PCR. The transcript level of *CmbHLH87* in the cultivar ‘JJJD’ at 0 h was used as control (quantities of calibrator) and assumed as 1. The values are the means ± SEs of three biological replicates. Data between treatments (112-2-treatment vs. 112-2-CK and JJJD-treatment vs. JJJD-CK) were analyzed by one-way ANOVA, and *denotes statistical significance at *p* < 0.05.

The *CmbHLH87* expression patterns in both PM-resistant inbred line ‘112-2’ and PM-susceptible cultivar ‘JJJD’ were analyzed in response to PM and exogenous treatments (H_2_O_2_, SA, ABA, MeJA, Eth, or NaCl) (**Figure 2**). The *CmbHLH87* expression level in the ‘112-2’ plants was significantly downregulated by PM (except at 0 hpi), but was significantly upregulated by H_2_O_2_ treatment (except at 6 hpi). In contrast, the *CmbHLH87* expression level in ‘JJJD’ plants was not significantly altered by PM, but was downregulated by H_2_O_2_ treatments. Regarding the effects of SA, the *CmbHLH87* expression level in ‘112-2’ plants decreased at 3 and 6 hpi, and was essentially unchanged thereafter. However, the *CmbHLH87* expression level in ‘JJJD’ plants was significantly upregulated by the SA treatment. In response to ABA, MeJA, Eth, and NaCl treatments, *CmbHLH87* expression was significantly higher in the ‘112-2’ and ‘JJJD’ seedlings than in the control (CK) seedlings. Specifically, the *CmbHLH87* expression level in ‘112-2’ plants was upregulated by more than 40-fold by Eth and NaCl treatments over the duration of the study period. The results indicated that *CmbHLH87* expression in PM-resistant inbred line ‘112-2’ was downregulated by PM and upregulated by H_2_O_2_, which differed from the results of the PM-sensitive cultivar ‘JJJD.’ Moreover, ABA, MeJA, Eth, and NaCl treatments obviously upregulated *CmbHLH87* expression in both analyzed plant materials and these were not influenced by susceptibility or resistance status.

### Improved PM Resistance of *CmbHLH87*-Overexpressing Tobacco Plants

**Figure 3:**
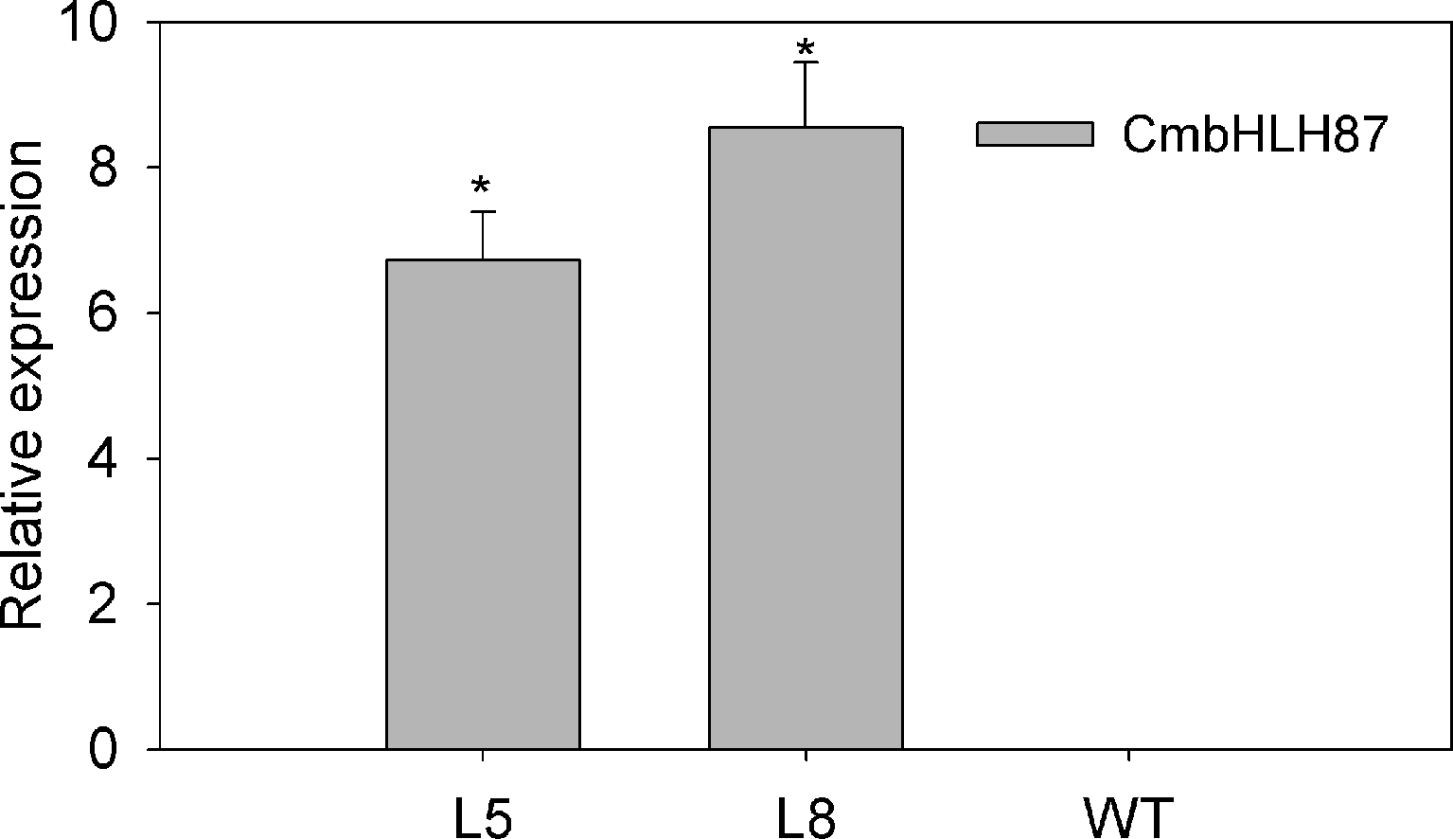
Relative expression of *CmbHLH87* in transgenic or wild-type plants under normal growth conditions. L5 and L8 are two independent transgenic lines that overexpress *CmbHLH87*. Three biological triplicates were averaged and bars indicate standard error of the mean. *denotes statistical significance at *p* < 0.05 between both materials.

**Figure 4:**
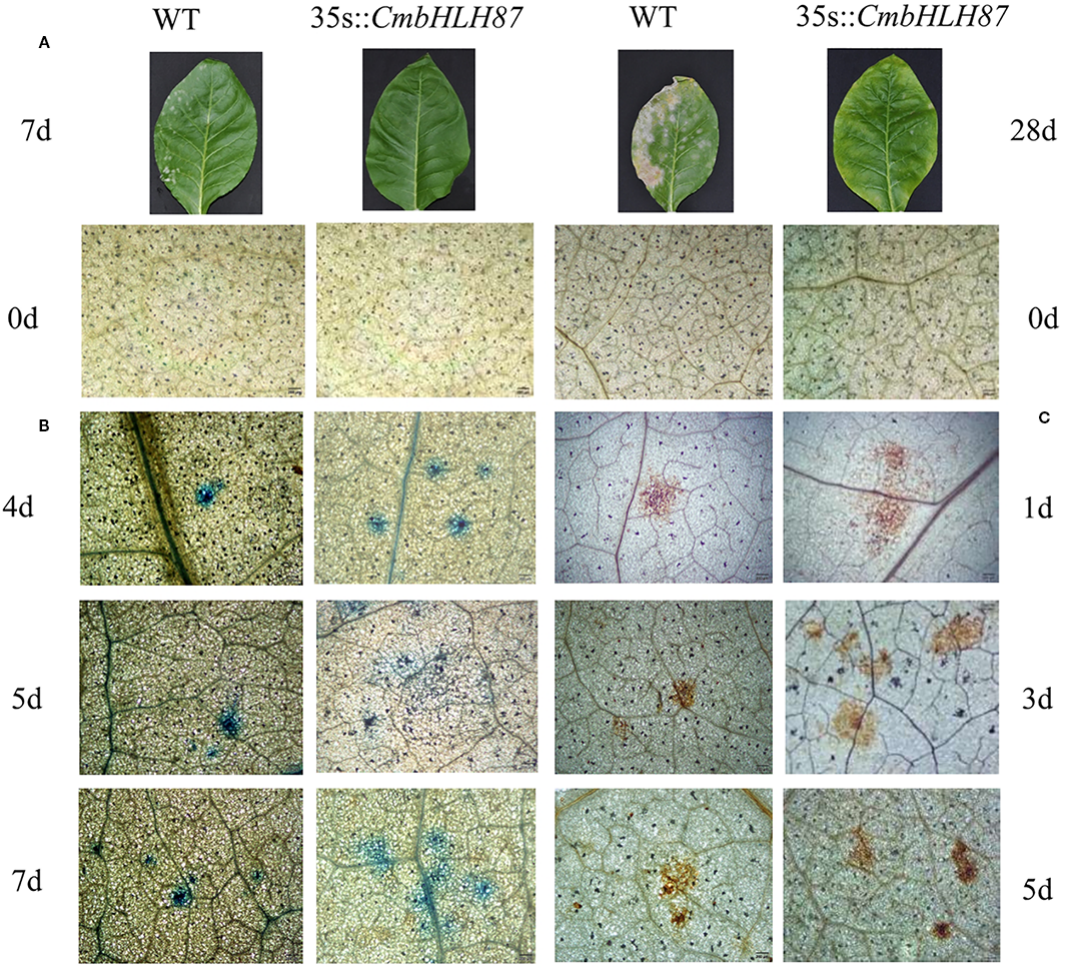
The pathogenic symptoms, trypan blue, and diaminobenzidine (DAB) staining of tobacco leaves infected with powdery mildew. The pathogenic symptoms of transgenic tobacco (L5) and wild type (WT) at 7 and 28 day post inoculation **(A)**; trypan blue staining was performed to visualize cell death **(B)**; DAB staining was performed to visualize hydrogen peroxide (H_2_O_2_) accumulation **(C)**. Scale bars = 200 μm.

The transcript level of the transgenic plants *CmbHLH87* under normal conditions was determined by qRT-PCR. The expression of *CmbHLH87* in wild-type (WT) plants was not basically examined, whereas the transgenic plants *CmbHLH87* expression was obviously upregulated (**Figure 3**), indicating that *CmbHLH87* is overexpressed in the transgenic plants. The disease severity (DS) of the transgenic plants was 84% lower than that of WT plants at 10 days postinoculation (dpi) (**Table 1**). Furthermore, the visible symptoms of leaf damage in tobacco seedlings were observed to examine the resistance of *CmbHLH87*-expressing plants to PM. As shown in **Figure 4A**, powdery symptoms were observed in WT seedlings at 7 dpi, and expanded considerably at 28 dpi, while transgenic plants exhibited undetectable after 7 dpi and slight powdery pots at 28 dpi. Blue spots are actually trypan blue stained cell death. The blue spots on the transgenic leaves ocurred at 4 dpi, continued to expand at 5 and 7 dpi, and those blue spots were more and bigger than those on the WT leaves, implying that overexpression of *CmbHLH87* in transgenic plants accelerated cell death following a PM infection (**Figure 4B**). Moreover, brown spots are H_2_O_2_ for DAB staining. Compared with WT leaves, there were more brown spots on the transgenic leaves at 1 dpi, larger and stained more intensely at 3 and 5 dpi. These results indicated that the overexpression of *CmbHLH87* promoted the accumulation of H_2_O_2_ in transgenic plants infected with PM (**Figure 4C**).

**Table 1:** Disease severity of leaves of tobacco seedlings infected with powdery mildew.

Materials	Disease severity (*in vitro* leaf)
L5 L8	6.50 ± 1.03 7.60 ± 1.01
L12 WT	8.00± 1.21 45.80± 1.41^*^

Data are mean values (± SD) of three independent experiments. *indicates significantly different values between treatments (p < 0.05). L5, L8, and L12 are three independent transgenic lines that overexpress CmbHLH87.

### Expression of Signal-Related Genes in Transgenic Tobacco Plants

**Figure 5:**
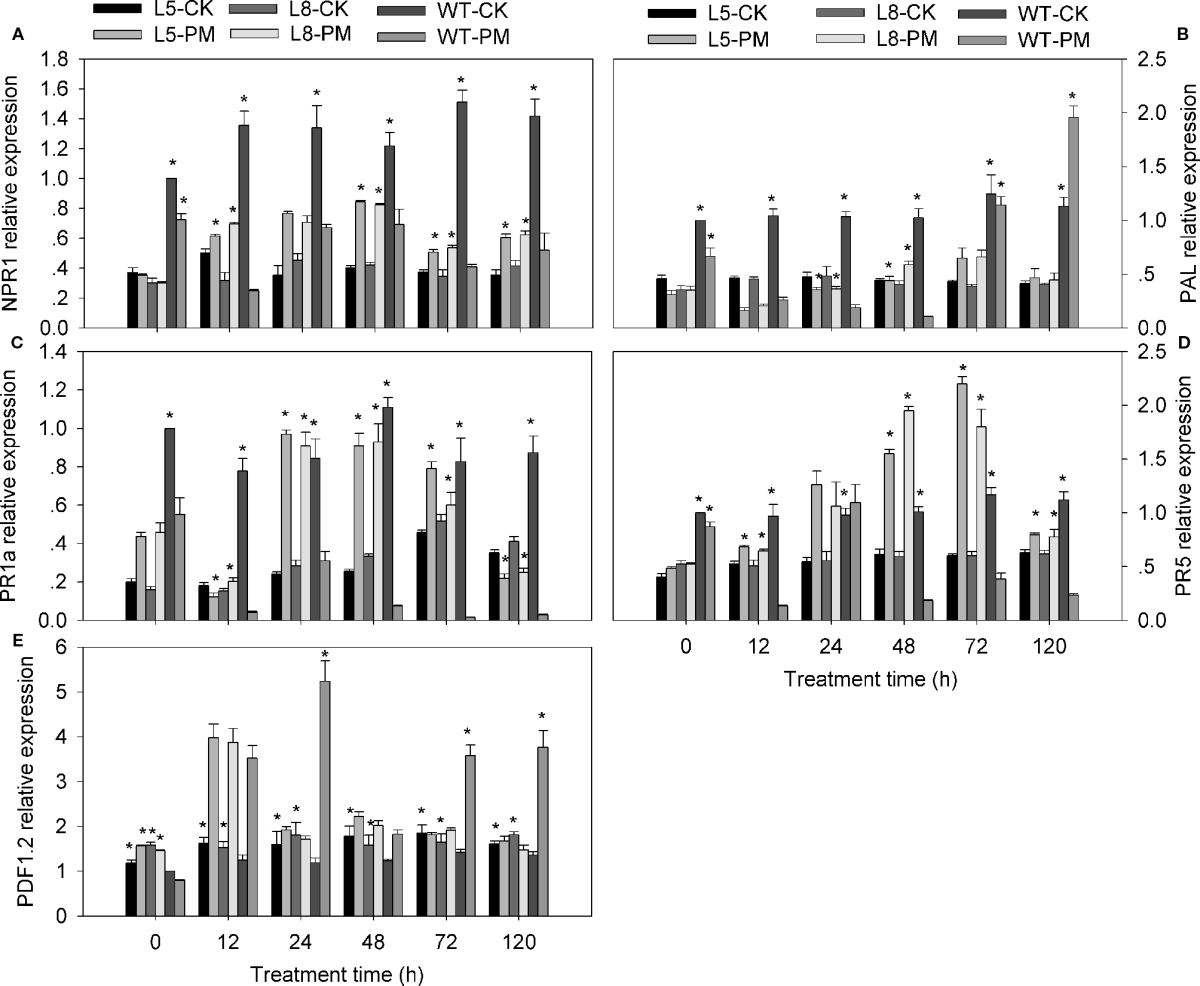
Expression of signal-related genes in transgenic and wild-type (WT) plants treated with powdery mildew. The samples of two genetically-modified tobacco lines (L5, L8) were used to analyze genes expression by qRT-PCR. A, *NtNPR1*; B, *NtPAL*; C, *NtPR1a*; D, *NtPR5*; E, *NtPDF1.2*. There were four treatments: L5/L8-CK represents transgenic lines under normal conditions; L5/L8-PM represents transgenic lines infected with powdery mildew; WT-CK represents WT plants under normal conditions; WT-PM represents WT plants infected with powdery mildew. Tobacco *NtEF1-α* gene (AF120093) was used as an internal reference. The expression levels of signal-related genes in WT plants at 0 h were used as control (quantities of calibrator) and assumed as 1. Three biological triplicates per line were averaged and Bars indicate standard error of the mean. Data between transgenic lines and WT plants (L5/L8-PM vs. WT-PM and L5/L8-CK vs. WT-CK) were analyzed by one-way ANOVA, and *denotes statistical significance at *p* < 0.05.

To investigate the signal transduction pathways affected by CmbHLH87 during plant defense responses to PM, the expression levels of five signaling-associated genes (*NPR1*, *PR5*, *PR1a*, *PAL*, and *PDF1.2*) in the SA, JA, and ET signal transduction pathways were analyzed by qRT-PCR (**Figure 5**). The *NPR1*, *PR1a*, and *PR5* expression levels were higher in the transgenic plants infected with PM than in the transgenic plants-CK plants, implying that PM induced the expression of these genes. And the opposite pattern of these genes expression was observed in the WT plants infected with PM compared with WT-CK plants. There were no basically differences in the *PAL* and *PDF1.2* (except at 12 hpi) expression levels of the transgenic plants between PM and CK treatments. In response to the PM infection, the *PR1a*, *PR5*, and *NPR1* expression levels of the transgenic plants were higher than those of the WT plants (except at 0 hpi). The *PAL* expression level in the transgenic plants was significantly higher at 24 and 48 hpi, lower at 12, 72, and 120 hpi than that in the WT plants. Furthermore, the *PDF1.2* expression level in the transgenic plants was slightly higher at 12 and 48 hpi, considerably lower at 24, 72, and 120 hpi than that in the WT plants. These results implied that *CmbHLH87* overexpression increased the transcription of *PR1a*, *PR5*, and *NPR1* and suppressed the expression of *PDF1.2* in transgenic plants infect with PM. Furthermore, the increased PM resistance of the transgenic plants appeared to be related to the upregulated expression of these genes.

### Improved Resistance of Transgenic Tobacco Plants to Bacterial Diseases

**Figure 6:**
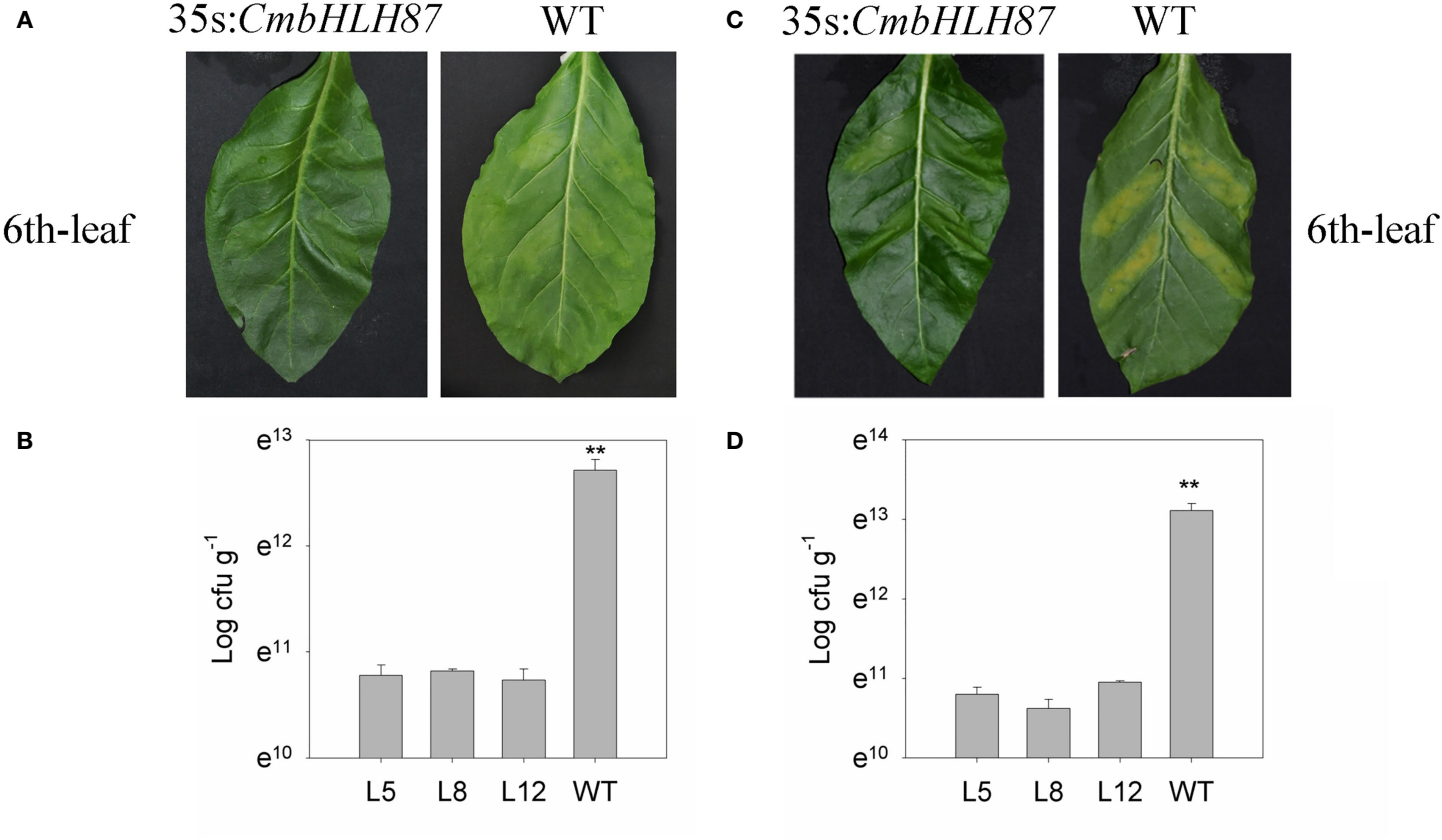
The resistance of *CmbHLH87* in tobacco plants to bacterial wilt and scab. **(A)** Pathogens symptoms of the 6th-upper leaf injection sites was injected with bacterial wilt bacteria with a needle-removed syringe; **(B)** concentration bacteria of the 6th-upper leaf injection sites was injected with bacterial wilt bacteria with a needle-removed syringe; **(C)** pathogens symptoms of the 6th-upper leaf injection sites was injected with scab bacteria with a needle-removed syringe; **(D)** concentration bacteria of the 6th-upper leaf injection sites was injected with scab bacteria. Three biological triplicates were averaged and bars indicate standard error of the mean. **denotes significant differences between wild-type (WT) and transgenic plants at *p* < 0.01.

To analyze the effect of CmbHLH87 on other plant diseases, two common bacterial pathogens causing bacterial wilt (*Ralstonia solanacearum*) and scab (*Xanthomonas euvesicatoria*) were injected into tobacco plants (**Figure 6**). At 6 dpi, the chlorosis and yellowing of the 6th leaf at injection sites were less severe for the transgenic plants than for the WT plants. Additionally, the concentrations of bacterial wilt and scab bacteria in the transgenic plants were 0.14 and 0.10 times those of the WT plants, respectively. These observations suggested that overexpression of *CmbHLH87* in tobacco increased the resistance to bacterial wilt and scab.

## Discussion

In this study, we isolated a novel pumpkin *bHLH87* gene and the predicted amino acid sequence included a conserved bHLH domain, suggesting that CmbHLH87 is a putative pumpkin bHLH transcription factor. Its predicted pI was 5.96, which is similar to a previous report that the isoelectric points of watermelon bHLH family proteins are mostly in the acidic range (He et al., 2016). The CmbHLH87 protein was localized to the nucleus in *Arabidopsis* protoplasts, which is consistent with the observations of earlier studies involving the bHLH transcription factors from other plant species (Wang et al., 2015).

The interplay among complex signaling networks, including various pathways regulated by phytohormones, such as SA, JA, ethylene (ET), and ABA, considerably influences plant resistance to diseases. An earlier investigation during an infection of wheat plants by the stripe rust pathogen infection, SA, MeJA, and particularly ET suppress the transcription of *TabHLH060* (Wang et al., 2015). In the current study, ABA, MeJA, Eth, and NaCl treatments induced pumpkin *CmbHLH87* expression, whereas PM infection had the opposite effect, suggesting that *CmbHLH87* may have a regulatory role during responses to hormones, salt stress, and PM. Additionally, overexpression of the pumpkin *CmbHLH87* gene in tobacco plants decreased the Disease severity by 84%, accelerated necrosis, and increased the accumulation of H_2_O_2_. These results indicated that the PM resistance of the transgenic tobacco plants was enhanced, likely because of the changes to the HR-related necrosis and H_2_O_2_ accumulation. Our findings are consistent with the results of an earlier investigation that H_2_O_2_ accumulation and subsequent cell death usually lead to the resistance to diseases caused by biotrophic pathogens (Li et al., 2011). The expression pattern of *CmbHLH87* in response to PM is controversial to its transgenic phenotype in disease resistance. There were similar reports that pathogen-regulated ectopic expression of bHLH transcription factors was inconsistent with activation of pathogen resistance. Two *Arabidopsis* bHLH25 and bHLH27 positively influence cyst nematode parasitism (*Heterodera schachtii*). Transgenic *Arabidopsis* plants overexpressing either one or both of the bHLH genes exhibited an increased susceptibility to *H. schachtii* (Jin et al., 2011). Jin et al. document an example of pathogen-induced ectopic coexpression of two regulatory genes to enhance pathogen success. In our experiment, this is also an intriguing biological phenomenon that highlights the complexity of obligate biotrophic plant–pathogen interactions who are direct target of the CmbHLH87 protein and how to interact in response to pathogen resistance need to be further research. PM symptoms herein were observed in WT tobacco seedlings at 7 dpi of *G. cichoracearum*, and the phenotypes were not more robust than those of previous reports: PM disease occurs abundantly in tobacco plants at 12 dpi of the PM strain *G. cichoracearum* SICAU1 (Li et al., 2019). Different tobacco accessions may show various susceptible PM disease phenotypes to different PM isolates.

SA plays a central role in plant disease resistance against biotrophic pathogens, while JA is critical for activating plant defenses against necrotrophic pathogens (Wasternack and Hause, 2013). *PDF1.2* is important for the JA/ET-dependent signaling pathway. At intermediate SA levels, NPR1 (nonexpresser of PR1) accumulates and interacts with the TGA transcription factor, functioning as a coactivator of SA-responsive genes, including *PR* genes (Caarls et al., 2015). The overexpression of wheat *TabHLH060* gene in *Arabidopsis* increases the susceptibility to *P. syringae* by suppressing the transcription levels of *PR1*, *PR2*, and *PR5*, which are involved in the SA signaling pathway, and by upregulating the expression of *PDF1.2* and *ORA59*, which are associated with the JA and ET signaling pathways (Wang et al., 2015). In bread wheat, *TaJAZ1* overexpression increases PM resistance by promoting the accumulation of reactive oxygen species. The encoded TaJAZ1 directly interacts with TaMYC4 (JA-induced bHLH transcription factor) to repress its transcriptional activity (Jing et al., 2019). In the current study, following PM infection, the *PR1a*, *PR5*, and *NPR1* expression levels in the transgenic plants were higher than those in the WT plants, whereas a different expression pattern was observed for the *PDF1.2* expression level. These results suggest that in the SA pathway, the transactivation of *PR1a*, *PR5*, and *NPR1* is dependent on CmbHLH87. Additionally, CmbHLH87 does not directly affect the JA/ET-dependent defense pathway to regulate *PDF1.2* expression. We propose that CmbHLH87 activates stress-resistance mechanisms *via* SA-dependent defense pathways without upregulating *PAL* expression, but suppresses the activities of JA/ET-dependent defense pathways. Therefore, we speculate that CmbHLH87 positively regulates the H_2_O_2_ and SA pathways. Moreover, H_2_O_2_ might directly transfer the SA signal to regulate the expression of downstream response genes in the *CmbHLH87*-overexpressing transgenic plants infected with PM. In NPR1-dependent SA signal transduction pathways, the activation of *PR* gene expression requires an interaction between NPR1 and the TGA transcription factor that binds to the target promoters (Fan and Dong, 2002; Spoel and Dong, 2012). We speculated whether there is a relationship between the upregulation of *NPR1* expression and SA-mediated transcriptional activation of *PR* genes. Notably, the phenotypes and genes influenced by *CmbHLH87* overexpression in tobacco plants might not be the same as those regulated by *CmbHLH87* expression in pumpkin in response to PM. Additional studies are necessary to reveal the biological functions of CmbHLH87 in pumpkin infected with PM.

Two globally important diseases that affect tobacco, bacterial wilt and scab, are caused by the necrotrophic *R. solanacearum* and the hemi-biotrophic *X. euvesicatoria* respectively. The overexpression of the bHLH transcription factor gene (*HBI1*) decreases the pathogen-associated defense responses and increases the susceptibility to bacteria *P. syringae* (Malinovsky et al., 2014). In the current study, the chlorosis and yellowing of the leaves near infection sites were less extensive in the transgenic plants than in the WT plants at 6 dpi. Moreover, the concentrations of bacterial wilt and scab bacteria were substantially lower in the transgenic plants than in the WT plants. These results imply overexpression of the pumpkin *CmbHLH87* gene in tobacco enhances the resistance to bacterial wilt and scab, which is consistent with the effects of *CmbHLH87* overexpression on the resistance to PM.

In conclusions, the results of this study indicate that overexpression of the pumpkin *CmbHLH87* gene in tobacco may increase the resistance to PM, bacterial wilt, and scab. Additionally, *CmbHLH87* overexpression may improve PM resistance by enhancing HR-related cell death and H_2_O_2_ accumulation and by upregulating the expression of SA-mediated defense genes. The data generated in this study may provide valuable genetic information for the breeding of new disease-resistant pumpkin varieties.

## Materials and Methods

### Plant Materials and Treatments

Pumpkin inbred line ‘112-2’ and cultivar ‘Jiujiangjiaoding’ (abbreviated ‘JJJD’), which are resistant and susceptible to PM, respectively, were provided by the Henan Institute of Science and Technology, Xinxiang, Henan, China (Zhou et al., 2010). Pumpkin seeds were germinated and the resulting seedlings were grown as previously described (Guo et al., 2018). Seedlings at the third-leaf stage were treated as previously described by Guo et al. (2019). Seedlings were sprayed with a freshly prepared spore suspension (10^6^ spores/ml), exogenous 1.5 mM H_2_O_2_, 100 μM SA, 100 μM ABA, 100 μM MeJA, 0.5 g/L Eth and 0.4mM NaCl. Moreover, water alone was used for the control treatment (CK). The treated seedlings were maintained in a growth chamber with a 15-h light (28°C)/9-h dark (18°C) cycle (5,500 lux light intensity) and harvested after 0, 3, 6, 12, 24, and 48 h to examine the *CmbHLH87* expression pattern. At each time point, two young leaves were collected from the upper parts of four seedlings (i.e., one sample), wrapped in foil, frozen in liquid nitrogen, and stored at −80°C. The treatments were arranged in a randomized complete block design, with three biological replicates.

### Isolation of *CmbHLH87* and Sequence Analysis

The *bHLH* homolog expressed sequence tag (GenBank accession number SRR5369792) was identified from a transcriptome of PM-resistant pumpkin seedling (Guo et al., 2018). Full length of this homolog was obtained using a homology-based candidate gene method (Guo et al., 2014). The theoretical molecular weight (Mw) and isoelectric point (pI) were calculated with the ExPASy Compute pI/Mw tool (Bjellqvist et al., 1993). Sequence data were analyzed with the ClustalW program (Thompson et al., 1994). The phylogenetic tree was constructed using Mega5.0 by the neighbor-joining method. The NCBI databases were screened for homologous sequences with the default parameters of the BLAST program (http://www.ncbi.nlm.nih.gov/blast) (Altschul et al., 1997).

### Subcellular Localization Analysis of CmbHLH87

The *CmbHLH87* ORF (without the termination codon) was ligated into the pBI221-GFP vector for the subsequent production of a green fluorescent protein (GFP)–tagged CmbHLH87 fusion protein. Polyethylene glycol was used during the transformation of *Arabidopsis thaliana* protoplasts with the recombinant plasmid (Lee et al., 2013). The subcellular localization of CmbHLH87 was determined based on the GFP signal, which was detected with the confocal fluorescence microscope (UltraVIEW VoX, Olympus, Japan) (Guo et al., 2019).

### Generation of *CmbHLH87* Transgenic Tobacco Plants

Pumpkin is known to be one of the plants most refractory for transformation. To date, only two reports on transformation in pumpkin existed using a combined method of vacuum infiltration and *Agrobacterium* infection (Nanasato et al., 2011; Nanasato and Tabei, 2015). So, we choosed an ectopic overexpression assay in tobacco instead of generating pumpkin transgenic plants. Forward and reverse primers with an added *Bam*H I site and *Kpn* I site, respectively, were used to amplify *CmbHLH87*. The amplified sequence was then inserted into the pVBG2307 vector for the subsequent expression of *CmbHLH87* under the control of the 35S cauliflower mosaic virus (CaMV) promoter. The recombinant plasmid was introduced into *Agrobacterium tumefaciens* GV3101 cells as previously described (Guo et al., 2014). The resulting *A. tumefaciens* cells were used to transform tobacco (*Nicotiana tabacum* L. cv. NC89) plants according to a previously described leaf disk method (Li et al., 2012). The transgenic plants were confirmed by examining the segregation ratio of the kanamycin selectable marker and by PCR analysis of *NPTII* and *CmbHLH87*, self-pollinated to obtain homozygous T2 offspring. T2 lines that produced 100% kanamycin-resistant plants in the T3 generation were considered as homozygous transformants. In each experiment, T2 generations of homozygous transgenic lines (L5, L8, and L12) were selected for further analysis. Similar phenotypes and results used for this study were observed in more than three independent lines of transgenic plants.

### Primer Design

All primers designed and used in this study are provided in **Supplementary Table S1**.

### Performance of Transgenic Lines Infected With PM, Bacterial Wilt and Scab

Conidia were collected from tobacco leaves naturally infected with *Golovinomyces cichoracearum*, which are main pathogen isolates of PM. The upper second leaf from the transgenic and WT seedlings at the fifth-leaf stage was sprayed with a spore suspension (10^6^ spores/ml) and contine to *in vitro* grow for 10 d (Guo et al., 2019). At 10 dpi, mildew development on each leaf disk was recorded, using the following scale: 0 = no visible mildew development, 1 = 0 to 5%, 2 = 6 to 25%, 3 = 26 to 50%, 4 = 51 to 75%, and 5 = > 76% of disk surface covered with mildew, as described by Ishii et al. (2001). Disease severity was calculated as [(5A + 4B + 3C + 2D + E)/5F] × 100, where A, B, C, D, and E were the number of leaf disks corresponding to the scales, 5, 4, 3, 2, and 1, respectively, and F was the total number of leaf disks assessed. Additionally, the second leaf of the transgenic and WT plants was sprayed with the abovementioned spore suspension and sampled at 0, 12, 24, 48, 72, and 120 hpi for a subsequent extraction of total RNA. Furthermore, these leaves were harvested symmetrically along the sides of the main vein after 0, 1, 3, 4, 5, and 7 d to examine cell death and H_2_O_2_ accumulation. 3, 3′ -diaminobenzidine (DAB) and trypan blue staining were used to analyze H_2_O_2_ accumulation and cell death, respectively, as previously described (Choi et al., 2012). The treatments were arranged in a randomized complete block design with three replicates.

The upper sixth leaf from the transgenic and WT seedlings at the twelfth-leaf stage were inoculated with bacterial solutions (10^8^ cfu/ml). The bacterial solutions were injected into the underside of leaves between the lateral veins with a syringe lacking a needle. After 6 d, the concentration of the bacteria at the injection sites was determined as previously described (Guo et al., 2019). The injection sites were sampled with a circular perforator (1cm diameter) and ground in aseptic water, then serially diluted to produce the 10^2^-fold, 10^3^-fold, and 10^4^-fold diluents. Experiments were done in triplicate for each line.

### qRT-PCR Analysis

The RNA extraction, first-strand cDNA synthesis and qRT-PCR were completed as described by Guo et al. (2015). Relative gene expression levels were determined with the 2^-ΔΔCT^ method. Total RNA was extracted from the leaves of pumpkin seedlings treated with various stresses or distilled water for 0, 3, 6, 12, 24, or 48 h as described above. The *β-actin* gene was used as an internal reference for normalizing of gene expression levels in pumpkin (Wu and Cao, 2010).

Total RNA was extracted from *CmbHLH87*-overexpressing and WT tobacco seedlings to examine the expression of five hormone-related genes (*NtNPR1*, *NtPR1a*, *NtPR5*, *NtPDF1.2*, and *NtPAL*) at 0, 12, 24, 48, 72, and 120 hpi as described above. The tobacco *NtEF1-α* gene (AF120093) was used as an internal control in the assays.

### Statistical Analyses

Values are herein provided as the mean ± standard error of three independent analyses. Data underwent a one-way ANOVA, and differences between WT and transgenic plants were evaluated with a *post hoc* comparison test (Student-Newman-Keuls method) at *p* < 0.05 with SPSS 19.0 for Windows (SPSS Inc, Chicago, IL).

## Data Availability Statement

The datasets generated for this study can be found in the the GenBank database (accession number MH105822).

## Author Contributions

W-LG and Y-YG conceived and designed the experiments. W-LG and B-HC performed the experiments. X-JC and Q-FL analyzed the data. H-LY, J-GZ, X-ZL, and W-LG contributed reagents/materials/analysis tools. W-LG wrote the paper. G-YW rectified the manuscript. All authors read and approved the manuscript.

## Funding

This research was funded by National Natural Science Foundation of China (Grant no. 31401876), Henan High-level Talent Introduction and Training Special Foundation (Grant no. 103020218003/008), the Henan Provincial commodity vegetable industry technology system (Grant no. S2010-03-G06).

## Conflict of Interest

The authors declare that the research was conducted in the absence of any commercial or financial relationships that could be construed as a potential conflict of interest.

## Supplementary material

Figure S1 A Neighbor-joining phylogenetic tree of CmbHLH87 and bHLH proteins from different plant species. The CmbHLH87 protein is marked with arrow. The bHLH subgroup names are shown to the right of square.

Figure S2 Amino acid sequences alignment of pumpkin CmbHLH87 with others. The conserved domains (bHLH) are shown by thin underlines. The genes included are bHLH87-like (*C. moschata* cv. ‘Rifu’, XP_022953326.1), bHLH87-like(*C. pepo*, XP_023547764.1), bHLH87-like (*C*. maxima, XP_022992322.1), bHLH87-like (*C. melo*, XP_008462383.1), bHLH87-like (*C. sativus*, XP_004141799.1), bHLH87-like (*Nicotiana tabacum*, XP_016504990.1), AtbHLH087 (*Arabidopsis*, AAM10960.1) and CmbHLH87 (*C. moschata* cv. ‘112-2’, MH105822).

Table S1 Primers used in this investigation.
