# Corrigendum: Expression of Pumpkin *CmbHLH87* Gene Improves Powdery Mildew Resistance in Tobacco

**DOI:** 10.3389/fpls.2021.779320

**Published:** 2021-12-09

**Authors:** Wei-Li Guo, Bi-Hua Chen, Yan-Yan Guo, Xue-Jin Chen, Qing-Fei Li, He-Lian Yang, Xin-Zheng Li, Jun-Guo Zhou, Guang-Yin Wang

**Affiliations:** ^1^School of Horticulture Landscape Architecture, Henan Institute of Science and Technology, Xinxiang, China; ^2^Henan Province Engineering Research Center of Horticultural Plant Resource Utilization and Germplasm Enhancement, Xinxiang, China

**Keywords:** pumpkin, powdery mildew, *CmbHLH87*, functional analysis, tobacco

In the original article, there was a mistake in Figure 6 as published. The leaf images in Figure 6A, second panel and Figure 6C, fourth panel are duplicated. The corrected Figure 6 appears below.

**Figure 6:**
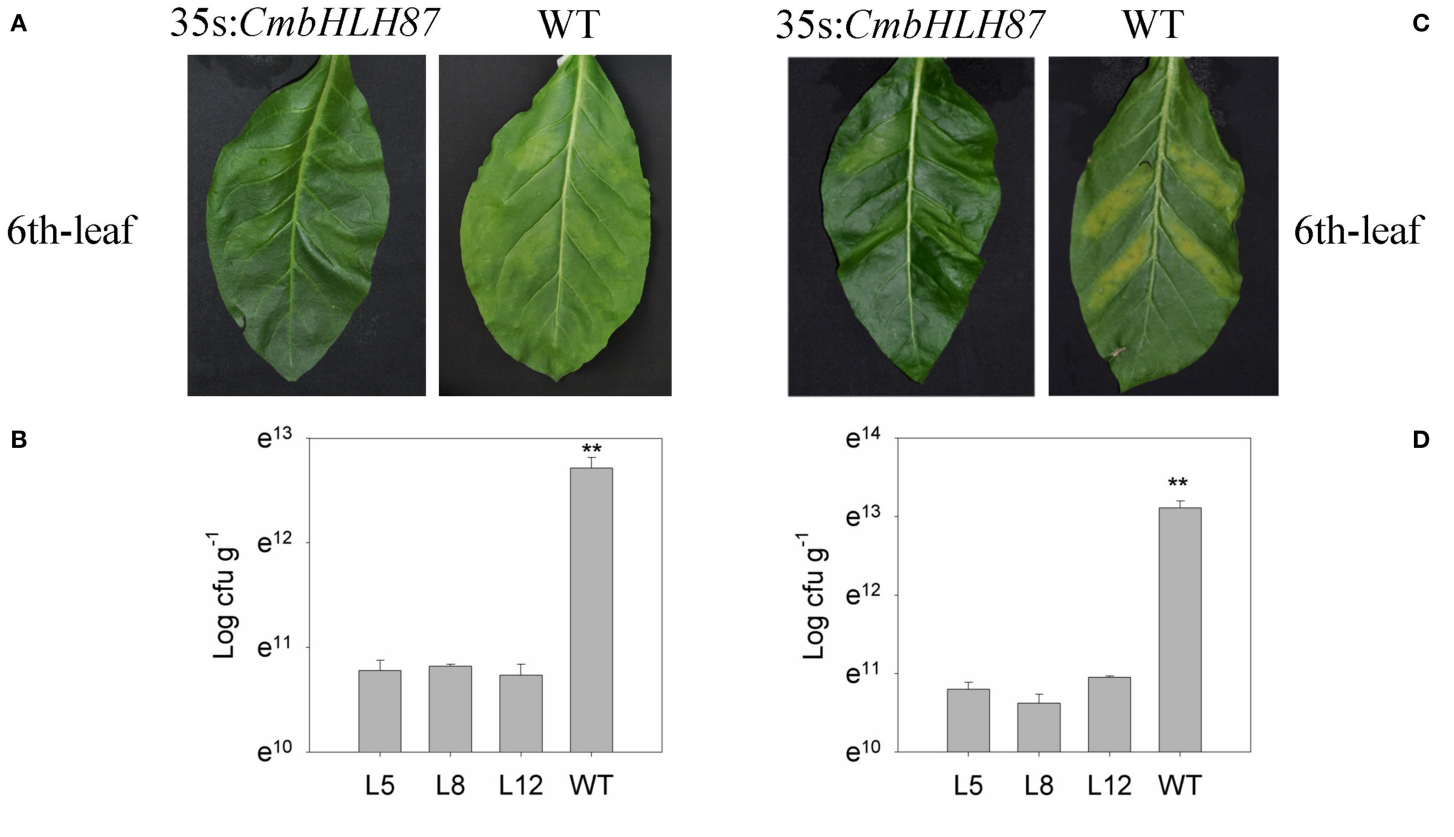
The resistance of CmbHLH87 in tobacco plants to bacterial wilt and scab. **(A)** Pathogens symptoms of the 6th-upper leaf injection sites was injected with bacterial wilt bacteria with a needle-removed syringe; **(B)** concentration bacteria of the 6th-upper leaf injection sites was injected with bacterial wilt bacteria with a needle-removed syringe; **(C)** pathogens symptoms of the 6th-upper leaf injection sites was injected with scab bacteria with a needle-removed syringe; **(D)** concentration bacteria of the 6th-upper leaf injection sites was injected with scab bacteria. Three biological triplicates were averaged and bars indicate standard error of the mean. ^**^denotes significant differences between wild-type (WT) and transgenic plants at *p* < 0.01.

The authors apologize for this error and state that this does not change the scientific conclusions of the article in any way. The original article has been updated.

## Publisher's Note

All claims expressed in this article are solely those of the authors and do not necessarily represent those of their affiliated organizations, or those of the publisher, the editors and the reviewers. Any product that may be evaluated in this article, or claim that may be made by its manufacturer, is not guaranteed or endorsed by the publisher.

