# Supplementary material for: Expression of Pumpkin CmbHLH87 Gene Improves Powdery Mildew Resistance in Tobacco
Source: Front Plant Sci. 2020 Apr 3;11:163. doi: 10.3389/fpls.2020.00163 (PMC7147351; doi:10.3389/fpls.2020.00163)
Supplement: Figure S1 — A Neighbor-joining phylogenetic tree of CmbHLH87 and bHLH proteins from different plant species. The CmbHLH87 protein is marked with arrow. The bHLH subgroup names are shown to the right of square. [file Image_1.pdf]

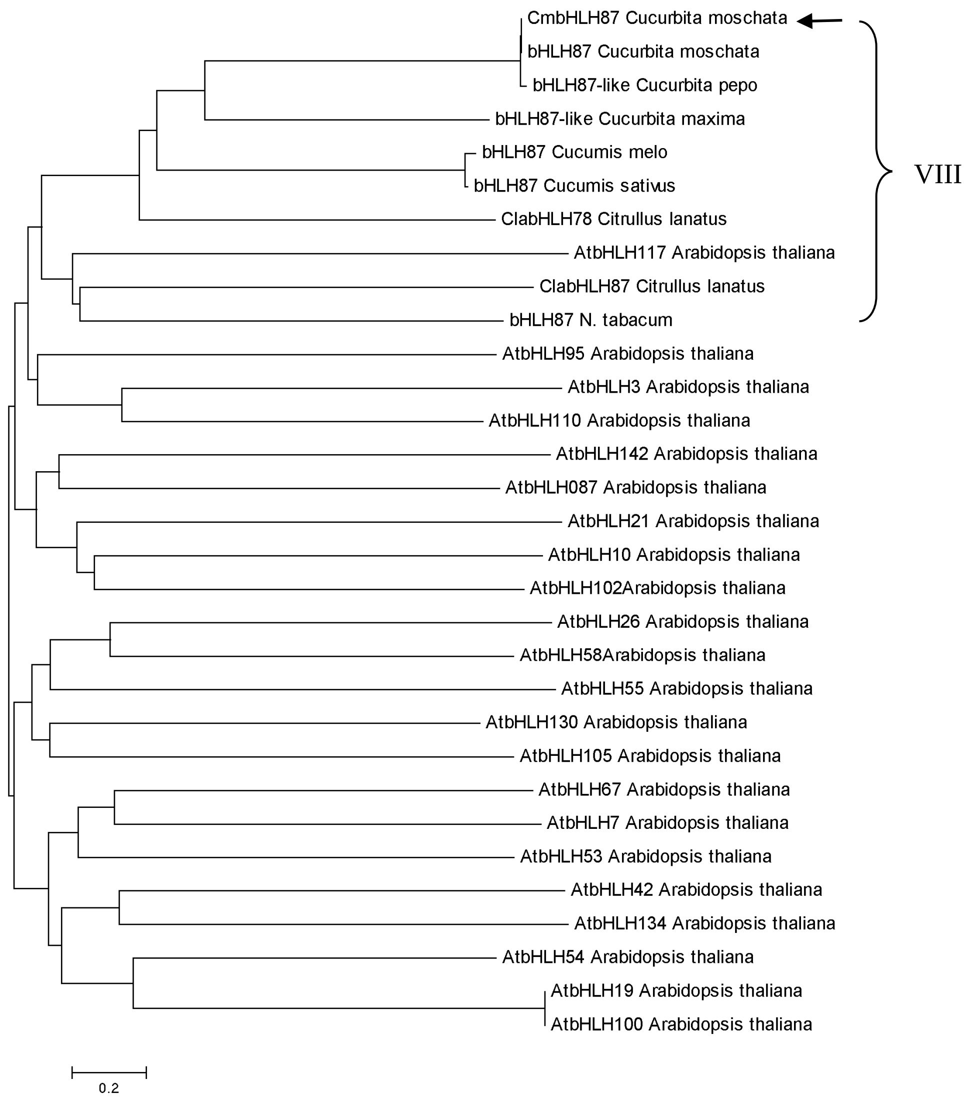

FIGURE S1 A Neighbor-joining phylogenetic tree of CmbHLH87 and bHLH proteins from different plant species. The CmbHLH87 protein is marked with arrow. The bHLH subgroup names are shown to the right of square.
