# Supplementary material for: Expression of Pumpkin CmbHLH87 Gene Improves Powdery Mildew Resistance in Tobacco
Source: Front Plant Sci. 2020 Apr 3;11:163. doi: 10.3389/fpls.2020.00163 (PMC7147351; doi:10.3389/fpls.2020.00163)
Supplement: Figure S2 — Amino acid sequences alignment of pumpkin CmbHLH87 with others. The conserved domains (bHLH) are shown by thin underlines. The genes included are bHLH87-like (C. moschata cv. ‘Rifu’, XP_022953326.1), bHLH87-like(C. pepo, XP_023547764.1), bHLH87-like (C. maxima, XP_022992322.1), bHLH87-like (C. melo, XP_008462383.1), bHLH87-like (C. sativus, XP_004141799.1), bHLH87-like (Nicotiana tabacum, XP_016504990.1), AtbHLH087 (Arabidopsis, AAM10960.1) and CmbHLH87 (C. moschata cv. ‘112-2’, MH105822). [file Image_2.pdf]

```
bHLH87-RIFU        MDHLSWDGYGSRATTNTPPPSSS..SFWSNYQQGHEQRFMISNNSNSPNFFNSVQDLP..    56
bHLH87-C. pepo     MDHLSWDGYGSRATTNTPPPSSS..SFWSNFQQGHEQRFMMSNNSNSLNFFNSVQDLP..    56
bHLH87-C. maxima   MDHLSWDGYGSRATTNTPPPPSSSLSFWSNYQQGHEQRFMISNNSNSPNFFNSVQDLP..    58
bHLH87-C._melo     MDHLNWNGYGSRVAKKTTASTTSSSSFWSNYQQGVEERFMISSSNNS.NFFNSVQDFPIK    59
bHLH87-C._sativu   MDHLNWNGYGSRVAKKTTASTTSSSSFWSNYQQGVEERFVISSSNNS.NFFNSVQDFPIK    59
bHLH87-N._tabacu   MESLNWD..ESPVFQEFGASFDP.IHVISNWNMPQRQEAAVRLAADS.MAAKSAADLSRD    56
AtbHLH087-A. tha   MEGLESVY..AQAMYGMTRESKIMEHQGSDLIWGGNELMARELCSSS.SYHHQLINPN..    55
CmbHLH87-112-2     MDHLSWDGYGSRATTNTPPPSSS..SFWSNYQQGHEQRFMISNNSNSPNFFNSVQDLP..    56
Consensus          m  l                        s                 s

bHLH87-RIFU        ...........................TSNMDVVGLETQLLTSFNGDKTATATAAT...C    86
bHLH87-C. pepo     ...........................TSNMDVVGLETQLLPSFNGDKTATATAATAATC    89
bHLH87-C. maxima   ...........................TSNMDVVGLETQLLTSFNGDKTATTTAAT...C    88
bHLH87-C._melo     VAENSSTPVLKTEDPLVMSPPLTSFTTSSNMDVVGFLSQLDHGSNGDKSATVTTCS....   115
bHLH87-C._sativu   VAANSSTPVLKTENPLAMSPSLTSFTTSSNMDVVGLLSQLDAGSNGDKSATVTTCS....   115
bHLH87-N._tabacu   CAENGFSSSPILNMPNYIS.......DSNPMALNGLMADFSSNGQVQEIKPAPKLLSNAC   109
AtbHLH087-A. tha   .........................LSSCFMSDLGVLGEIQQQHVGNRASSIDPS....    86
CmbHLH87-112-2     ...........................TSNMDVVGLETQLLTSFNGDKTATATAATAATC    89
Consensus                                        m   g

bHLH87-RIFU        SLESLDCLLSATN..SNTDTSIEDDGSVSMMLSDYTNLWNFGGNAAP..SSK........   134
bHLH87-C. pepo     SLESLDCLLSATN..SNTDTSIEDDGSVSMMLSDYTNLWNFGGNAAP..SSK........   137
bHLH87-C. maxima   SLESLDCLLSATN..SNTDTSIEDDGSVSMMLSDYTNLWNFGGNAAP..SSK........   136
bHLH87-C._melo     .LESLDCLLSATN..SNTDTSVEDDGSVSMMLTDYTNLWNFGGNAAV..SSKESEKNG..   168
bHLH87-C._sativu   .LESLDCLLSATN..SNTDTSVEDDGSVSMMLTDYTNLWNFGGNAAV..SSKESEKNG..   168
bHLH87-N._tabacu   SLESLDCLLSATTTTNNTDTSIEDDGMSMIFVDNSKGLWNFNSSSEHNASSEALKQSLGY   169
AtbHLH087-A. tha   ...SLDCLLSATS..NSNNTSTEDDEGISVLFSDCQTLWSFGGVSSAESENREITTETTT   141
CmbHLH87-112-2     SLESLDCLLSATN..SNTDTSIEDDGSVSMMLSDYTNLWNFGGNAAP..SSK........   137
Consensus             sldcllsat       ts edd            lw f

bHLH87-RIFU        ...........................................RSHDQTHIKPTEYSVFS   151
bHLH87-C. pepo     ...........................................RSHDQTHIKPTEYSVFS   154
bHLH87-C. maxima   ...........................................RSHDQTHIKPTEYSVFS   153
bHLH87-C._melo     ......................................SNSTKRSHEQTQFKAADYSIFS   190
bHLH87-C._sativu   ......................................SNSTKRSHEQTQFKAADYSIFS   190
bHLH87-N._tabacu   QQLLTCHESGHKILQEEERKRKPYKPINANELDETVSQSSPNHKYNKRSHNQTLENSPSE   229
AtbHLH087-A. tha   TIKP....................KPLKRNRGGDGGTTETTTTTTKPKSLKRNRGDETGS   181
CmbHLH87-112-2     ...........................................RSHDQTHIKPTEYSVFS   154
Consensus

bHLH87-RIFU        NNIITLSDSTSNSEGFKIITDQNLPKQKKPR......SEKPPNSSNINFQQSCSSGSSCI   205
bHLH87-C. pepo     NNIITLSDSTSNSEGFKIITDQNLPKQKKPR......SEKPPNSSNINFQQSCSSGSSCI   208
bHLH87-C. maxima   NNIITLSDSTSNSEGFKIITDQNLPKQKKPR......SEKPPNSSNINFQQSCSSGSSCI   207
bHLH87-C._melo     NNIINLSDSTSDSGGFRIITDHNLPKQKKPR......SEKPPSSSNINFQQSCSSGSSCI   244
bHLH87-C._sativu   NNIINLSDSTSDSGGFRIITDHDLPKQKKPR......SEKPPSSSNINFQQSCSSGSSCI   244
bHLH87-N._tabacu   FNLFESDSLNGDCGNFQLISEK.QSKSKKPRLIAENYNKSRPISSNINFQQASSS.VSSI   287
AtbHLH087-A. tha   HFSLVHPQDDSEKGGFKLIYDENQSKSKKPR.....TEKERGGSSNISFQHSTCL...SD   233
CmbHLH87-112-2     NNIITLSDSTSNSEGFKIITDQNLPKQKKPR......SEKPPNSSNINFQQSCSSGSSCI   208
Consensus                         f  i      k kkpr            ssni fq

bHLH87-RIFU        DQEPDPEAIAQMKEMIYRAAAFRPVNLGLEAIEKPKRKNVRISTDPQTVAARQRRERISE   265
bHLH87-C. pepo     DQEPDPEAIAQMKEMIYRAAAFRPVNLGLEAIEKPKRKNVRISTDPQTVAARQRRERISE   268
bHLH87-C. maxima   DQEPDPEAIAQMKEMIYRAAAFRPVNLGLETIEKPKRKNVRISTDPQTVAARQRRERISE   267
bHLH87-C._melo     DQEPDPEAIAQMKEMIYRAAAFRPVNLGLEMIEKPKRKNVRISTDPQTVAARQRRERISE   304
bHLH87-C._sativu   DQEPDPEAIAQMKEMIYRAAAFRPVNLGLEMIEKPKRKNVRISSDPQTVAARQRRERISE   304
bHLH87-N._tabacu   DQEPDPEAIAQMKEMIYRAAAFRPVNFGPEAPEKPKRKNVRISTDPQTVAARQRRERISE   347
AtbHLH087-A. tha   NVEPDAEAIAQMKEMIYRAAAFRPVNFGLEIVEKPKRKNVKISTDPQTVAARQRRERISE   293
CmbHLH87-112-2     DQEPDPEAIAQMKEMIYRAAAFRPVNLGLEAIEKPKRKNVRISTDPQTVAARQRRERISE   268
Consensus            epd eaiaqmkemiyraaafrpvn g e  ekpkrknv is dpqtvaarqrrerise

bHLH87-RIFU        RIRVLQRIVPGGSKMDTASMLDEAANYLKFLKSQIKALENLGQKIESLNCPSNTIAFSFN   325
bHLH87-C. pepo     RIRVLQRIVPGGSKMDTASMLDEAANYLKFLKSQIKALENLGQKIESLNCPSNTIAFSFN   328
bHLH87-C. maxima   RIRVLQRIVPGGSKMDTASMLDEAANYLKFLKSQIKALENLGQKIESLNCPSNSIAFSFN   327
bHLH87-C._melo     RIRVLQRLVPGGSKMDTASMLDEAANYLKFLKSQIKALENLGQKLESLNCPPTSIAFSFN   364
bHLH87-C._sativu   RIRVLQRLVPGGSKMDTASMLDEAANYLKFLKSQIKALENLGQKLESLNCPPTSIAFSFN   364
bHLH87-N._tabacu   RIRVLQRLVPGGSKMDTASMLDEAANYLKFLRSQVEALEAIGQKQD....PFSSIQFNY.   402
AtbHLH087-A. tha   KIRVLQTLVPGGTKMDTASMLDEAANYLKFLRAQVKALENLRPKLD.....QTNLSFSSA   348
CmbHLH87-112-2     RIRVLQRIVPGGSKMDTASMLDEAANYLKFLKSQIKALENLGQKIESLNCPSNTIAFSFN   328
Consensus           irvlq  vpgg kmdtasmldeaanylkfl  q  ale    k            f

bHLH87-RIFU        P.SFPIQTSSSSSSHNNFSLQNPYDQLM.....                              352
bHLH87-C. pepo     P.SFPIQTSSSSSSHNNFSLQNPYDQLM.....                              355
bHLH87-C. maxima   P.SFPIQTSSSSSSHNNFSLQNPYDQLM.....                              354
bHLH87-C._melo     P.SFPIQTSS...SHNNFTLLNPNHLINQYPQN                              393
bHLH87-C._sativu   P.SFPIQTSS...SHNNFTLLNPNHLINQYPQN                              393
bHLH87-N._tabacu   ..PFPMQLPH.......FPVQNPNPIHGPKS..                              424
AtbHLH087-A. tha   PTSFPLFHPS......FLPLQNPNQIHHPEC..                              373
CmbHLH87-112-2     P.SFPIQTSSSSSSHNNFSLQNPYDQLM.....                              355
Consensus             fp                np
```

FIGURE S2 Amino acid sequences alignment of pumpkin CmbHLH87 with others. The conserved domains (bHLH) are shown by thin underlines. The genes included are

bHLH87-like (*C. moschata* cv. ‘Rifu’, XP_022953326.1), bHLH87-like (*C. pepo*, XP_023547764.1), bHLH87-like (*C. maxima*, XP_022992322.1), bHLH87-like (*C. melo*, XP_008462383.1), bHLH87-like (*C. sativus*, XP_004141799.1), bHLH87-like (*Nicotiana tabacum*, XP_016504990.1), AtbHLH087 (*Arabidopsis*, AAM10960.1) and CmbHLH87 (*C. moschata* cv. ‘112- 2’, MH105822).
