# Supplementary material for: Expression of Pumpkin CmbHLH87 Gene Improves Powdery Mildew Resistance in Tobacco
Source: Front Plant Sci. 2020 Apr 3;11:163. doi: 10.3389/fpls.2020.00163 (PMC7147351; doi:10.3389/fpls.2020.00163)
Supplement: Table S1 — Primers used in this investigation. [file Table_1.docx]

Table S1 Primers used in this investigation

| Gene | Accession | Primer sequence(5’-3’) |
| --- | --- | --- |
| *CmbHLH87* | MH105822 | F: TTTGTCATCGGTTCGGTAAG  R: GACTCCCGTTCGTTCTCAAG |
| RT-qPCR for *Cm bHLH87* |  | F: CAAACTCATATCAAACCAACAGA  R: TATCATCTCCTTCATTTGTGCTA |
| Over-  expression  vector |  | F:GGGGATCCTTTGTCATCGGTTCGGTAAG (*BamH* I)  R:GGGGTACCGACTCCCGTTCGTTCTCAAG ( *Kpn* I) |
| *NtNPR1* | U76707 | F: ACATCAGCGGAAGCAGTAG  R: GTCGGCGAAGTAGTCAAAC |
| *NtPR1a* |  | F: CCTCGTACATTCTCATGGTCAAT  R: CCATTGTTACACTGAACCCTAGC |
| *NtPR5* |  | F: CCGAGGTAATTGTGAGACTGGAG  R: CCTGATTGGGTTGATTAAGTGCA |
| *NtPDF1.2* | T04323 | F: GGAAATGGCAAACTCCATGCG  R: ATCCTTCGGTCAGACAAACG |
| *NtPAL* | X95342 | F: GTTATGCTCTTAGAACGTCGCCC  R: CCGTGTAATGCCTTGTTTCTTGA |
| *NtEF1-α* | AF120093 | F: TGTGATGTTTTTGTTCGGTCTTTAA  R: TCAAAAGAAAATGCAGACAGACTCA |
| *β-actin*  *NPTII* |  | F: TCTCTATGCCAGTGGTCGTA  R: CCTCAGGACAACGGAATC  F: AGACAATCGGCTGCTCTGAT  R: TCATTTCGAACCCCAGAGTC |
